# Resveratrol Acts Not through Anti-Aggregative Pathways but Mainly via Its Scavenging Properties against Aβ and Aβ-Metal Complexes Toxicity

**DOI:** 10.1371/journal.pone.0021565

**Published:** 2011-06-27

**Authors:** Alberto Granzotto, Paolo Zatta

**Affiliations:** Centro Nazionale delle Ricerche, Istituto Tecnologie Biomediche (CNR-ITB), Metalloproteins Unit, Department of Biology, University of Padova, Padova, Italy; Semmelweis University, Hungary

## Abstract

It has been recently suggested that resveratrol can be effective in slowing down Alzheimer's disease (AD) development. As reported in many biochemical studies, resveratrol seems to exert its neuro-protective role through inhibition of β-amyloid aggregation (Aβ), by scavenging oxidants and exerting anti-inflammatory activities. In this paper, we demonstrate that resveratrol is cytoprotective in human neuroblastoma cells exposed to Aβ and or to Aβ-metal complex. Our findings suggest that resveratrol acts not through anti-aggregative pathways but mainly via its scavenging properties.

## Introduction

Alzheimer's disease (AD) is one of the most common form of dementia worldwide. Although the underlying causes of AD are still debated, two pathological hallmarks have been identified: senile plaques (SPs) and neurofibrillary tangles (NTFs). The latter are formed by hyperphosphorilation and abnormal deposition of tau (τ) protein. SPs consist of deposits of β-amyloid protein (Aβ) mainly. Aβ derives from proteolitical cleavage of the amyloid precursor protein (APP) by three enzymes: α-, β- and γ-secretase. When APP is metabolized by β- and γ-secretase, Aβ_1–40_ and the more toxic form Aβ_1–42_ are produced; a phenomenon that is known as the “amyloidogenic pathway" [1]. An imbalance between production and clearance of these aggregative prone peptides triggers the formation of SPs [2]. Even though SPs are the most evident AD hallmark, recent reports highlight that Aβ oligomers, because of their potent synaptotoxicity, play a crucial role in AD onset and development [3]–[5].

This scenario is further complicated by a huge amount of variables that can influence Aβ aggregation pathway and toxicity, such as the dyshomeostasis of brain metal ions [6]–[8]. As a matter of fact brain metal dismetabolism has been widely demonstrated in AD patients and it has been proposed as a potential etiological co-factor [9]–[11]. Accordingly to this idea, metals accumulation in the elderly could be seen as a risk factor for AD onset and development. The unbalanced presence of metal ions in the brain can easily exacerbate the oxidative properties of Aβ [12]–[14] and its toxicity [15], [16].

A mechanism used by Aβ, in the presence of metal ions, to exert its toxicity is the production of reactive oxygen species (ROSs).

Several natural compounds have been proposed to date to reduce the oxidative stress found in AD brains [17]–[19]. Among these compounds, resveratrol provoked great interest. Resveratrol is a natural polyphenol widely present in plants and in particular in the skin of red grapes and in wine; resveratrol antioxidant properties have been well demonstrated [20], with a wide range of biological effects [21], and fortunately, the compound is free of adverse effects [22]. In addition, recent papers underline its Aβ anti-aggregative properties [23]–[26]. Despite all these positive effects, a major constraint holding back the use of resveratrol is its poor bioavailability when taken as dietary supplement [27].

The aim of this study is to test whether resveratrol might have anti-amyloidogenic and fibril-destabilizing properties, not only just against Aβ but also against Aβ-metal complexes and to assess whether the compound can act as a neuroprotectant. To that aim, we employed neuroblastoma cell cultures treated with Aβ complexes in presence or absence of resveratrol.

## Results

### Congo Red assay

**Figure 1:**
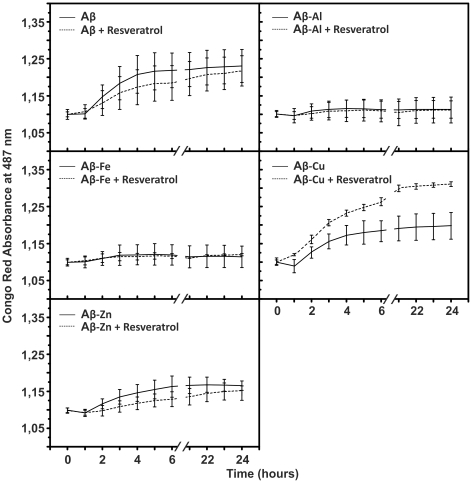
Congo Red spectroscopic assay. Time-dependence of Congo Red (CR) absorption when CR is bound to Aβ and to Aβ-metal complexes, in the presence and in the absence of resveratrol. The reaction mixtures containing 1,5 µM Aβ or Aβ-metal complexes, 0.1 M Tris/HCl, 0.15 M NaCl, pH 7,4 and 0 (solid line) or 45 µM (dash) resveratrol, were incubated at room temperature for the indicated times. The absorbance due to CR was subtracted. Each point represents means ± SD of three individual experiments.

Congo Red (CR) is largely used in histochemical studies to detect Aβ fibril deposits. Accordingly to Nilsson (2004) [28], CR can be also used to investigate amyloid fibrillization *in vitro*. As shown in Fig. 1, with the exception of the Aβ-Cu metal complex, resveratrol had no effect on the fibrillization of other Aβ metal complexes. On the contrary, the presence of resveratrol seemed to enhance the propensity of the Aβ-Cu complex to form fibrils. Strikingly, Aβ-Al and Aβ-Fe showed no or little propensity to aggregate compared with Aβ alone and its complexes with Cu and Zn. These results are not surprising, as we have previously hypothesized, and recently largely confirmed, that Al is able to “freeze" Aβ in its oligomeric state, stabilizing this assembly [29], [30].

### Turbidity measurements

**Figure 2:**
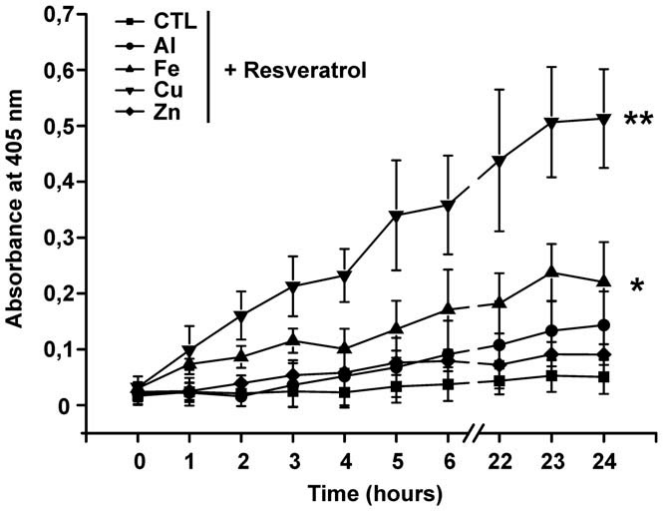
Turbidity assay. Turbidity assay of resveratrol in the presence of Al, Fe, Cu and Zn. Each well contained 200 µM resveratrol and 400 µM metal ions. The experiment was carried out in Tris/HCl buffer, as described in Materials and methods. The absorbance due to metallic solutions was subtracted. Data represented are mean ± SD of three independent experiments. * P<0,05; ** P<0,01.

To define a possible interaction between metal ions and resveratrol, turbidity measurements of resveratrol in the presence of metal ions (Al, Fe, Cu and Zn) were performed. An increase in absorbance value at 405 nm is indicative of the ability of resveratrol to form aggregates. Absorbance values over time are shown in Fig. 2. As reported in literature, resveratrol shows a strong propensity to form complexes with Cu *in vitro* [20]; in agreement with these data, solutions containing resveratrol and Cu showed a significant increase in absorbance. Our data also confirm the ability of resveratrol to form complexes with Fe and to a lesser extent with Al and Zn.

### Cell viability assay

**Figure 3:**
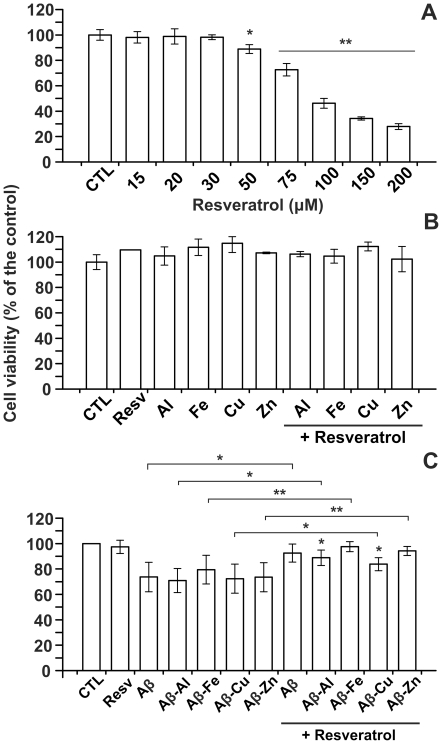
Cytotoxicity assay in neuroblastoma cells. The dependence of neurotoxicity (% cell death compared with the control) on the concentration of resveratrol (A) is shown. To exclude neurotoxicity due to the interaction between resveratrol and metal ions, SH-SY5Y cells were incubated for 24 hours with Al, Fe, Cu and Zn (5 µM) in the presence and in the absence of resveratrol (15 µM); in this case no significant toxicity was observed (B). Fig. C shows cell viability after treatment with Aβ and Aβ-metal complexes (0,5 µM) with or without resveratrol (15 µM). Results were obtained in four individual experiments. Error bar indicate the mean ± SD. * P<0,05; ** P<0,01.

Before testing the effect of resveratrol on neuroblastoma cells exposed to Aβ and or its metal complexes, a toxicity profile of the compound was performed and cell death evaluated by MTT assay. The resveratrol concentration needed to inhibit 50% (IC_50_) of cell viability was 100 µM (Fig. 3A). Resveratrol at 15 µM proved to be largely non toxic and was thereby chosen for the experiments in which neuroblastoma cells were exposed to Aβ, metal ions, or Aβ-metal complexes.

As shown in Fig. 3B, resveratrol was not toxic when mixed with metals (15 µM for resveratrol and 5 µM for metal ions).

As largely demonstrated by this laboratory [15], [30], Aβ-Al was the most effective in reducing cell viability on neuroblastoma cells when compared with Aβ alone or other Aβ-metal complexes. In the presence of resveratrol there was a significant decline in cells mortality. Treatment with resveratrol resulted in significant neuroprotection against Aβ and Aβ-metal induced toxicity. Resveratrol drastically reduced the toxicity triggered by Aβ-Fe and Aβ-Zn (p<0.01), while seemed less effective on Aβ, Aβ-Al and Aβ-Cu-induced cell death (p<0.05; Fig. 3C). These results have some limitations due to the use of a trasformed cell line (neuroblastoma, SH-SY5Y). Despite this, data obtained could be helpful to fix the basis for a follow up study.

### TEM

**Figure 4:**
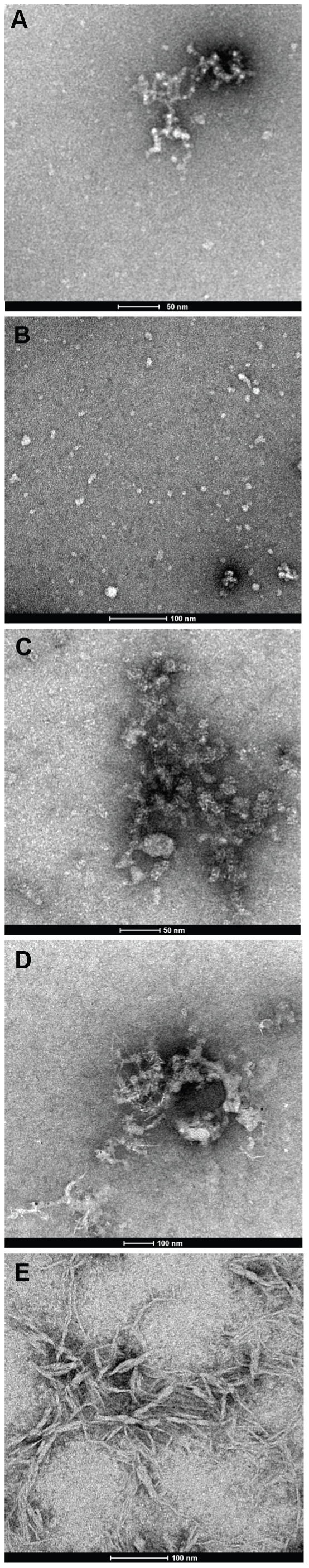
TEM micrographs. TEM micrographs of Aβ and Aβ-metal complexes in the presence of resveratrol after 24 hours of incubation at room temperature. The final protein concentration was 10 µM, while resveratrol concentration was 300 µM (molar ratio 1∶30). A) Aβ+resveratrol; B) Aβ-Al+resveratrol; C) Aβ-Fe+resveratrol; D) Aβ-Cu+resveratrol; E) Aβ-Zn+resveratrol.

With TEM investigated and compared the morphology of Aβ and the Aβ metal aggregates with resveratrol after 24 hours of incubation. The results are consistent with the CR assay. Aβ retained the ability to form mature protofibrils in the presence of resveratrol (Fig. 4A), likewise Aβ-Al retained its oligomeric structure (Fig. 4B). It is worth noting that the CR assay confirmed what we have previously shown with other biophysical techniques (ThT fluorescence and TEM) [15], [30]. Aβ-Cu and Aβ-Zn formed unstructured aggregates both in the presence and absence of resveratrol (Fig. 4D and 4E respectively). Unlike the results obtained during the CR assay, Aβ-Fe showed the propensity to form unstructured fibrils similar to those found in the case of Aβ-Cu and Aβ-Zn (Fig. 4C).

### SOD assay

**Figure 5:**
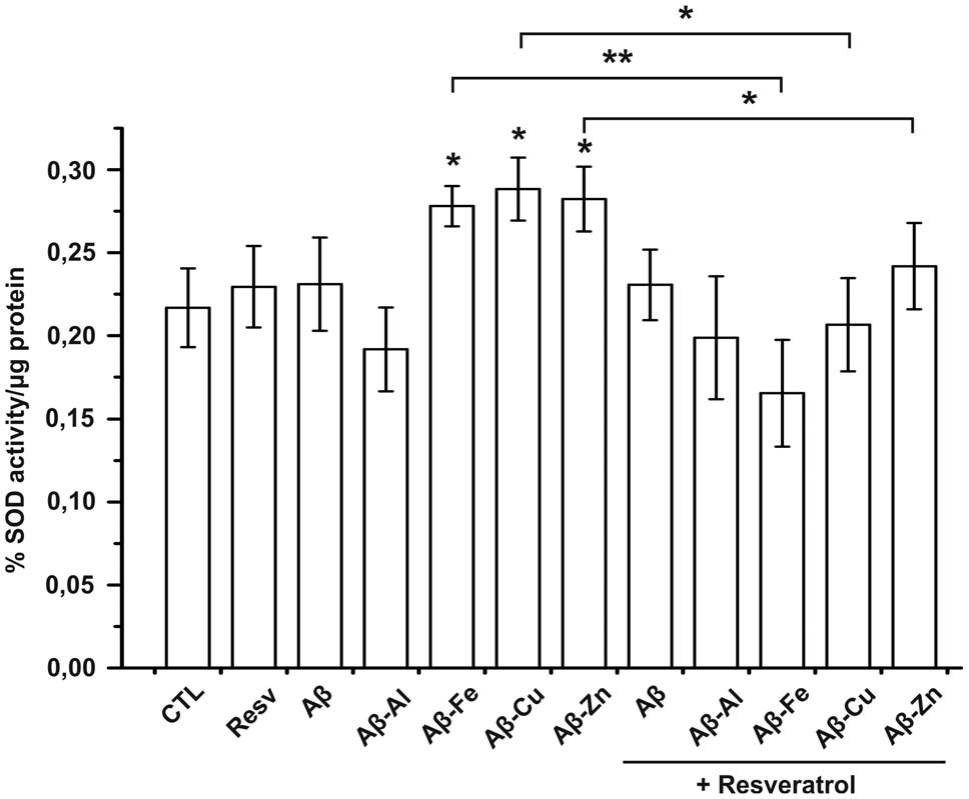
SOD activity assay. SOD activity was measured on cells treated with Aβ and Aβ-metal ions, with or without resveratrol. Concentrations were the same used for MTT assay. Results were obtained in four individual experiments. Error bar indicate the mean ± SD. * P<0,05; ** P<0,01.

Finally, we tested the effect of Aβ and Aβ-metal complexes on SOD activity in the presence or absence of resveratrol. 24 h treatments with Aβ-Fe, Aβ-Cu and Aβ-Zn caused a significant increase in SOD activity (p<0.05), while Aβ and Aβ-Al showed negligible effects, even though Aβ-Al complex seemed to reduce SOD activity but not in a statistically significant manner. Resveratrol was able to revert this process as we observed a decrease in SOD activity in samples containing Aβ-Fe, Aβ-Cu or Aβ-Zn, suggesting that the compound has anti-oxidant properties. As for Aβ and Aβ-Al we did not observe significant changes in SOD expression in the presence of resveratrol (Fig. 5B). SOD activity was also tested after treating neuroblastoma cells with metal ions alone (with or without resveratrol), to rule out that the SOD increase was merely due to the presence of transition metals. A large excess of Al, Fe, Cu and Zn ions was used (5 µM) in these control experiments and we did not observed significant and reproducible changes (data not shown).

## Discussion

It has been reported that resveratrol can extend the lifespan in several organisms [31]–[33] and therefore the compound has gathered great interest as anti-aging molecule.

In our study, it is demonstrated how resveratrol can reduce the toxicity in neuroblastoma cells exposed to either Aβ or Aβ-metal complexes. We chose Aβ-metal complexes because metal ions (such as Al, Cu, Fe and Zn) greatly potentiate Aβ aggregation as well as its intrinsic toxicity [15], [30].

Several papers have highlighted that resveratrol can be a potent anti-amyloidogenic and fibril-destabilizing polyphenol [23]–[26]. In our opinion this neuroprotective mechanism of action is unsatisfactory for two reasons: 1) in accordance with the observations reported by Hudson et al. [34] resveratrol biases the Thioflavin T fluorescence assay for amyloid fibril detection through nonspectral interferences. 2) In the context of AD, anti-aggregative drugs might exert more harm than as amyloid oligomers are more toxic than fibrils [35], [36]. We performed our CR assay to detect the presence of amyloid fibrils in the presence of resveratrol and found (Fig. 1), that the compound does not influence Aβ-metal complexes aggregative pathway, except for Aβ-Cu where we observed an increase in fibrillization. One possible explanation could be that resveratrol stabilizes Aβ-Cu complex in more ordered structures because of its Cu chelating properties (Fig. 2). In agreement with the CR assay, TEM micrographs do not show an anti-amyloidogenic effect of resveratrol.

After excluding an anti-amyloidogenic activity observed during our experimental conditions (see different Aβ procedure preparation in [26]), we set to investigate any potential antioxidant properties. It has been widely demonstrated that the AD brain shows damages caused by ROS [37]–[39]. In AD, ROS are the byproduct of several pathological events, including the production of hydrogen peroxide by Aβ [40]–[42] and the accumulation of transition metals (such as Fe^3+^, Cu^2+^ and Zn^2+^) [43]–[45]. Aβ-metals complexes cause the coexistence of these two by-products, exacerbating damages due to cellular oxidative stress. In accordance with this hypothesis, we observed an increase in super-oxide dismutase (SOD) activity in neuroblastoma cells treated with Aβ-Fe, Aβ-Cu and Aβ-Zn compared with non treated cells (Fig. 5B). SOD (both as SOD1 and SOD2) act as an antioxidant, protecting cells from being damaged by free radical species [46]; however, their increased level is an indication of cellular oxidative stress due to ROS overproduction. Neuroblastoma cells treated simultaneously with Aβ-metal complexes and resveratrol showed negligible differences in SOD activity when compared with relevant controls. Altogether these data suggest that Aβ-Fe and Aβ-Zn complexes exerted their toxicity mainly through oxidative stress; while Aβ-Cu seemed to exert its toxicity through different pathways; in fact this complex was still toxic, even in the presence of resveratrol.

Aβ and especially Aβ-Al resulted significantly toxic on neuroblastoma cells, a phenomenon that occurred without increasing SOD activity, suggesting that Aβ and Aβ-Al were not directly involved in free radical species production in our experimental conditions. In this connection, Al^3+^ is neither a redox metal nor involved in oxidative stress processes [47]–[49], meanwhile Aβ, not complexed with metals, is involved in of H_2_O_2_ production but it seems not involved in that of free radical species [40]. Diversely, Aβ and Al capacity to produce free radicals is linked exclusively to the presence of transition metals such as Fe and Cu [9], [41], [45], [47], [50], [51]. Nevertheless, resveratrol promoted neuroprotection also against Aβ and Aβ-Al-mediated toxicity suggesting that it can act through alternative mechanisms that do not require SOD activity. It is noteworthy to point out that resveratrol plays its neuroprotective role through several activities including: activation of protein kinase C, reduction of malondialdehyde levels, blocking of COX-2 expression, reduction of neuroinflammatory responses and scavenging [20], [25], [52]–[54].

Despite these chemopreventive properties, Aβ-Al and Aβ-Cu retained toxic activity on neuroblastoma cells even after treatment with resveratrol; this suggests that these two Aβ-metal complexes exert their toxicity through mechanisms that cannot be prevented by resveratrol. These findings are in agreement with previous data from our lab indicating that Aβ-Al can damage cell membranes because of its high superficial hydrophobicity [16]. Similarly, Aβ-Cu exerts its toxicity through mechanisms that are not only oxidative stress dependent [55], [56].

Collectively, our findings indicate that : 1) in our experimental conditions we did not observe any anti-amyloidogenic and fibril-destibilizing effect played by resveratrol, as proposed by other groups [23]–[26], [54], [57]; 2) resveratrol exerts its neuroprotective activity not only against Aβ but also against Aβ-metal complexes; 3) resveratrol acts as a ROS scavenger against those generated by Aβ-Fe, Aβ-Cu and Aβ-Zn, thereby reducing their toxicity; and 4) eventually, resveratrol is not sufficient to fully block Aβ-Al and Aβ-Cu toxicity.

## Materials and Methods

### Materials

Human β-amyloid 1–42 was purchased from Invitrogen. L-lactic acid aluminum salt, FeCl_3_, CuCl_2_, ZnCl_2_, 3-(4,5-dimethylthiazol-2-yl)-2,5-diphenyltetrazolium bromide (MTT) and resveratrol were purchased from Sigma-Aldrich (St. Louis, Mo.). Congo Red was purchased from Merck & Co., Inc (Whitehouse Station, N.J.). All *in vitro* experiments were carried out in 0.1 M Tris/HCl pH 7.4 buffer plus 0.15 M NaCl (standard medium).

### Preparation of Aβ-metal complexes

Human Aβ was solved in hexafluorisopropanol (HFIP) for 40 min at room temperature. HFIP was removed under vacuum in a Speed Vac (Sc110 Savant Instruments). This treatment was repeated three times (modified protocol from [58]). The Aβ metal complexes were prepared by 24-h dialysis against metal solutions 10 mM ([CH_3_CH(OH)COO]_3_Al, FeCl_3_, CuCl_2_, ZnCl_2_) at T = 4°C using Spectra/Por® Float-A-Lyser® tubes (Spectrum Labs) with 100 Molecular Weight Cut Offs (MWCO). Then, Aβ metal complexes were dialyzed against bidistilled water (three water changes, pH = 7) for 24 h to remove the excess of metals. The same treatment was also performed with Aβ alone. Aliquotes of Aβ, Aβ-metal complexes were stored at −20°C until used.

### Congo Red spectroscopy assay

Congo Red (CR) spectroscopic assay was performed in agreement with Nilsson's protocol (2004) using a 300 µL 96-well plate with U-bottom [28]. Kinetic was followed for 24 h by monitoring the changes in absorbance at 487 nm using a Microplate SPECTRAmax® reader. The increase in absorbance at this wavelength is indicative of amyloid fibrils formation. The final protein concentration in each well was 1,5 µM, while resveratrol concentration was 45 µM (concentration ratio protein-resveratrol was 1∶30). Resveratrol was dissolved in absolute ethanol (final concentration 100 mM) and further diluited as needed. The final ethanol concentration in wells was 2% (v/v). This concentration of ethanol in solution did not change Aβ and Aβ-metal complexes aggregation kinetics (data not shown). The signals due to the buffer alone was subtracted.

The absorbance of a solution containing resveratrol, metal ions (Al, Fe, Cu and Zn) and CR dye was measured at two wavelengths: 405 nm and 487 nm, to exclude potential artifactual cross-interactions. The first wavelength to exclude the formation of precipitates (turbidity assay), the second to exclude the capability of resveratrol and metal ions to coordinate CR. Concentrations in each well were: as for CR and resveratrol the same used in Aβ fibrils detection (70 µg/ml and 15 µM respectively), while for metal ions 3 µM. Results obtained allow us to state that resveratrol and metal ions did not seem to bias CR spectroscopic activity (data not shown).

### Turbidity measurements

Turbidity assay was performed using a 300 µL 96-well plate with flat bottom. Absorbance at 405 nm was read using a Microplate SPECTRAmax® reader. The concentrations in the wells were the following: resveratrol 200 µM, metals (Al, Fe, Cu, Zn) 400 µM, ethanol 2%. The absorbance due only to metallic solutions was subtracted. Resveratrol only kinetics is reported to exclude possible hydrophobic interaction between the molecules in solution causing precipitation; resveratrol is sparingly soluble in water (solubility 0.03 g/L). Data reported are not biased by spectroscopic interferences due to resveratrol, its UV spectrum (not shown) shows a maximum at 308 nm [20], while all turbidity measurements were carried out at 405 nm.

### Transmission Electron Microscopy (TEM)

All samples at 10 µM protein concentration, after an incubation period of 24 h, were absorbed onto glow-discharged carbon-coated butwar films on 400-mesh copper grids. The grids were negatively stained with 1% uranyl acetate and observed at 40,000× by transmission electron microscopy (TEM) (Tecnai G2, FEI). The samples observed contained Aβ and its metal complexes with resveratrol 300 µM in 2% v/v of absolute ethanol.

### Neuroblastoma Cells

SH-SY5Y human neuroblastoma cells were purchased from ECACC (European Collection of Cell Culture, Salisbury, UK). The medium in which they were cultured contained DMEM/F12 (Gibco, Carlsbad,CA USA) with 15% (v/v) fetal bovine serum (FBS, Sigma-Aldrich, St. Loius, MO), 100 units/ml penicillin and 100 µg/ml streptomycin (Gibco, Carlsbad, CA USA) and 1% (v/v) MEM non essential amino acid (NEAA) (Sigma-Aldrich, St. Loius, MO). Cells were stored at 37°C with 5% CO_2_ in a humidified atmosphere (90% humidity). Cells were used until passage 25 for both the MTT assay and the SOD assay. The culture medium was replaced every two days.

### Cell Viability Assay

Cell viability was determined through MTT reduction assay. SH-SY5Y cells were seeded into 24-well plates at a density of 15×10^4^ cells per well in 1 ml culture medium. 15% FBS-culture medium containing: Aβ, Aβ-metal complexes (0.5 µM) with or without resveratrol (15 µM) was added to the cells for 24 hours. Resveratrol was dissolved in absolute ethanol, the final ethanol concentration in the medium was 0,2% (v/v). This ethanol concentration resulted largely non-toxic (data not shown). 100 µL of 5 mg/ml MTT was added to each well and incubated in the dark at 37°C for 3 hours. Then cells were lysed with 1 ml of acidic isopropanol (0.04 M HCl in absolute isopropanol) [59]. Color intensity was measured with a 96-well ELISA plate reader at 550 nm (Microplate SPECTRAmax®). Toxicity due to metals alone (5 µM) in the presence and in the absence of resveratrol (15 µM) was also tested. All MTT essays were performed three times, in triplicate. Viability was defined as the relative absorbance of treated *vs.* untreated, expressed as a percentage.

### SOD assay

Total cellular superoxide dismutase (SOD) activity was determined with a SOD assay kit (Sigma-Aldrich) by following manufacturer's protocol. 1.0×10^6^ neuroblastoma cells were seeded into 25 cm^2^ flasks. Cells were grown at 80% confluency, then cells were treated with Aβ and Aβ-metal complexes (0.5 µM in the medium) in the presence and in the absence of resveratrol (15 µM in the medium). After 24 h cells were scraped, washed three times in cold PBS buffer and then disrupted using Cell Extraction Buffer (Invitrogen) containing 1 mM PMSF and 1× protease inhibitor cocktail (Sigma-Aldrich). Whole cell lysates of SH-SY5Y was centrifuged at 10,000 rpm (4°C, 10 minutes). Supernatant was transferred into 0.5 mL Eppendorf tube. Obtained solutions were used to determine protein concentration and SOD activity.

### Statistical Analysis

Congo Red spectroscopy, MTT, turbidity and SOD assays were statistically analyzed by Student's *t* test and one-way analysis of variance. Results were reported as highly statistically significant if P<0,01 and statistically significant if P<0,05. Results are presented as mean ± standard deviation (SD).
